# Visual Diagnosis: Pediatric Airway Emergency

**DOI:** 10.1155/2012/495363

**Published:** 2012-03-27

**Authors:** Bobby Desai, Lars Beattie, Matthew F. Ryan, Michael Falgiani

**Affiliations:** Department of Emergency Medicine, University of Florida College of Medicine, Gainesville, FL 32608, USA

## Abstract

We present a case of a potentially difficult airway emergency in a pediatric patient. After presentation, we briefly review critical differences between pediatric and adult airways and management of the airway during these emergencies.

## 1. Case Presentation

A 4-year-old girl was brought to the Emergency Department (ED) by her parents with an impaled foreign object in her mouth. The parents stated that she was running with a wire hanger in her mouth when she subsequently tripped, fell, and had the hanger lodge into the back of her throat. Her mother noted she had no significant medical history, and the event had occurred less than 20 minutes prior to arrival to the ED. The mother stated the patient had been able to speak after the event but was able to grunt responses to simple questions. The patient was awake and alert and could nod appropriately to yes-no questions.

Upon arrival, the patient was quite agitated, and vital signs were deferred due to her condition. Airway equipment and medications for rapid sequence intubation were emergently brought to the bedside. Respiratory therapy was also present to prepare for potential jet ventilation. Soon after arrival, the patient became increasingly agitated, and at this time, Otolaryngology and Anesthesia Departments were asked to evaluate the patient at bedside. The emergency physician, otolaryngology attending and anesthesia attending, all felt that due to her increasing agitation it would be best to secure her airway in the operating arena. She was subsequently transferred to the operating room, where she was successfully intubated and the foreign was body removed. She had no complications, and was discharged after a twenty-four-hour observation period.

## 2. Discussion

The pediatric airway and respiratory function differ from those in adults; the differences in the anatomy between adults and infants are particularly marked [1]. For example, the head shape is completely different in infants compared with older children and adults, as the occiput is more protuberant. Other notable anatomic differences in infants include a relatively large tongue in relationship to the mouth, a longer, thinner, and thus less compliant epiglottis, and a larynx that lies more anterior and caudal resulting in a shorter thyromental distance. The larynx is typically described as being cone-shaped with the narrowest segment at the level of the cricoid cartilage [2]. The cricoid is a complete ring of cartilage, so any mucosal edema at this level will encroach on the lumen resulting in exponential increases in resistance to air flow per Poiseuille's law, that is, air flow is proportional to the fourth power of the airway radius.

Developmental changes in the soft-tissue structures of the upper airway occur with age. Radiographic studies show that bony structures remain proportionately the same size. Adenoidal tissue disproportionately increases in the size between 3 and 5 years of age, resulting in a narrowing of the nasopharyngeal airway. Subsequently, bony growth outstrips soft tissue growth, and the airway dimensions increase [3]. Therefore, due to these anatomic differences when muscle tone is reduced, for example, in the setting of a reduced level of consciousness, the head will flex and pharyngeal tone will diminish, therefore, resulting in reduced oropharyngeal volume and occlusion of the oropharynx by the tongue. Accordingly, airway-opening maneuvers are required to maintain airway patency. Here, the application of basic adult principles is usually sufficient to provide airway support until additional pediatric help is available. Simple airway-opening techniques, such as head tilt and jaw thrust, are usually sufficient to open the child's airway [4].

Differences in pulmonary physiology also affect airway management. Infants have higher oxygen consumption rates (6–8 mL/kg/min versus 4–6 mL/kg/min) than adults. Infants also have a higher ratio of minute ventilation to functional residual capacity. This results in steep declines in arterial oxygen partial pressures if the airway becomes occluded and subsequently requires more rapid resolution of airway compromise I hypoxic injury is to be avoided [5].

**Figure 1:**
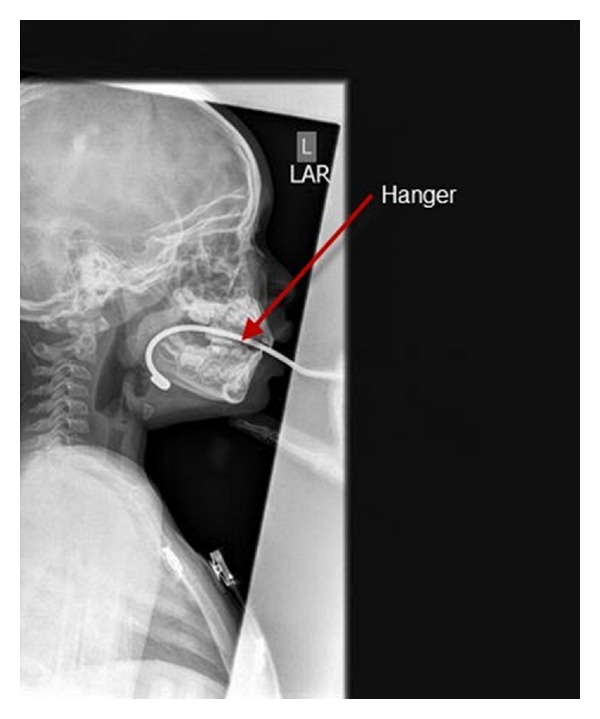
Lateral neck X-ray showing foreign body.

**Figure 2:**
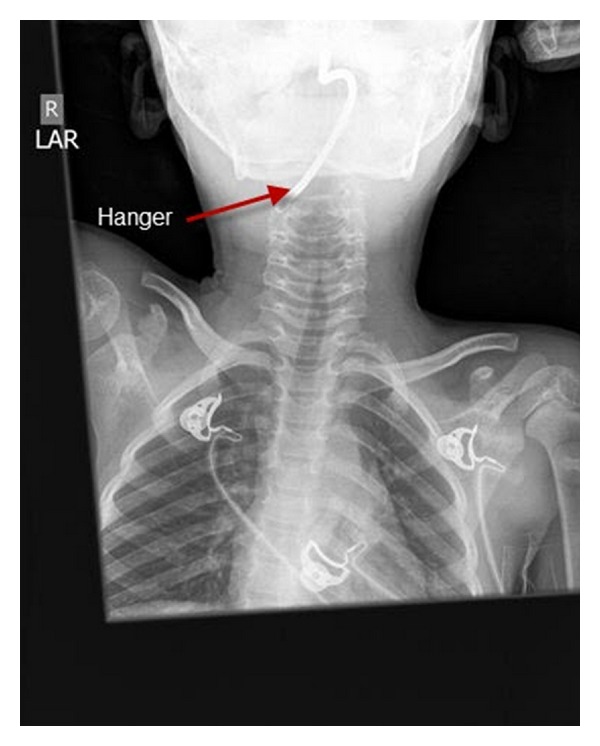
Anterior/Posterior neck X-ray again showing foreign body.

In unconscious children, a variety of techniques can be used to open the airway. In those who have spontaneous respiratory effort, *lateral positioning *may be all that is required. Lateral positioning also improves airway patency in unconscious children who have their airway maintained with simple airway maneuvers. However, it is not clear what the optimum simple airway maneuver in children is. Jaw thrust appears superior to simple positioning in the “sniffing” position. Studies in anesthetized children have mixed results. One study shows chin lift as equally effective as open-mouth jaw thrust in improving airway patency, while other reports show jaw thrust to be superior. Both maneuvers apply anterior tension to the hyoid bone, and they draw the epiglottis away from the posterior pharyngeal wall, opening the pharynx. In addition, the jaw thrust pulls the tongue away from the palate and opens the oropharynx. Thus, only the jaw thrust opens both the pharynx and oropharynx. Moreover, the jaw thrust maneuver can be a potent arousal stimulus, also improving respiratory effort. Care must be taken to use the minimum amount of head tilt necessary to open the airway (Figures 1 and 2) [1].

When is it appropriate to intubate versus using a bag-valve-mask for ventilation? A well-designed, prospective controlled trial comparing bag-mask ventilation with tracheal intubation in the out-of-hospital setting found no difference in outcome in 830 children randomized between these two techniques. Of particular concern, however, subgroup analysis found that survival was worse for intubated children in the respiratory failure/arrest group. Fifteen children in the tracheal intubation group had unrecognized esophageal intubation or tracheal tube dislodgement, and all but one of these died. Furthermore, a study of outcomes and complications in a series of 31,464 children who had severe head injury showed no advantage of tracheal intubation. Finally, an examination of survival predictors in pediatric trauma patients showed that prehospital tracheal intubation was an independent predictor of poor outcome. Some authors, therefore, suggest that the gold standard for initial airway management in children should be bag-mask ventilation with or without airway adjuncts of oropharyngeal and nasopharyngeal airways [6].

When all other attempts have failed, needle cricothyrotomy is the last resort. There are several potential problems with this technique in children. The cricothyroid membrane is far less well defined than in adults, particularly in infants, and the cricoid ring is the narrowest part of the airway. Thus, a needle placed just below the thyroid cartilage may not bypass an airway obstruction. In practice, it may be easier and more effective to insert a needle between two prominent upper tracheal rings. The largest catheter over-needle system that will pass should be used (e.g., 14 gauge). Spontaneous ventilation will not be possible through this catheter. Ventilation must be supported because positive pressure ventilation through the catheter does not maintain normocarbia, and oxygenation is the only realistic goal. The connector from a 3.0 mm (or 3.5 depending on the make) tracheal tube will connect to the Luer lock of the IV cannula and can be used to connect to a self-inflating bag. Alternatively, if a continuous oxygen source is available, the tubing can be connected to a three-way stopcock (with all lumens open), which is in turn attached to the cannula. With a flow rate of 1 L/min/year of age, intermittent occlusion of the open port of the tap will insufflate gas into the trachea. This latter technique carries a high risk of barotrauma. Commercial insufflation devices that provide Luer-lock connections and medication ports are available. Cricothyrotomy is rarely performed. There is no data supporting its use in children, although there are anecdotal accounts of individual successes [7].

## Abbreviations

ENT: Ear, Nose, and Throat
IV: Intravenous.
